# Development and validation of a combined ultrasound-pathology model to predict axillary status after neoadjuvant systemic therapy in breast cancer

**DOI:** 10.7150/ijms.101855

**Published:** 2024-10-21

**Authors:** Wenjie Shi, Jinzhi He, Xuan Li, Hailing Zha, Rui Chen, Lu Xu, Xiaoming Zha, Jue Wang

**Affiliations:** 1 Department of Breast, Women's Hospital of Nanjing Medical University, Nanjing Maternity and Child Health Care Hospital, No. 123 Tianfei Street, Mochou Road, Nanjing, 210004, China.; 2 Department of Breast Disease, the First Affiliated Hospital of Nanjing Medical University, No.300 Guangzhou Road, Nanjing, 210000, China.; 3 Department of Ultrasound, the First Affiliated Hospital of Nanjing Medical University, No.300 Guangzhou Road, Nanjing, 210000, China.; 4 Department of Clinical Nutrition, the First Affiliated Hospital of Nanjing Medical University, No.300 Guangzhou Road, Nanjing, 210000, China.

**Keywords:** breast cancer, ultrasound, axillary lymph nodes, pathological complete response, nomogram

## Abstract

**Background:** This study aimed to develop a combined ultrasound (US)-pathology model to predict the axillary status more accurately after NST in breast cancer.

**Methods:** This retrospective study included breast cancer patients who received NST at the First Affiliated Hospital of Nanjing Medical University from 2015 to 2022. Clinical, US, and pathological data were collected. Univariate and multivariate logistic regression analyses were performed to identify independent predictors of axillary pathological complete response (pCR). The model was developed using the predictors and validated.

**Results:** A total of 657 patients were enrolled in this study. Two multivariate logistic analyses were performed before and after the operation. The results showed that the clinical lymph nodes, ER status, HER2 status, chemotherapy response of primary tumor, hilum structure of axillary lymph nodes (ALNs) after NST, blood flow of ALNs after NST, vascular invasion, pathological size, and Miller-Payne grade (all p < 0.05) were independent predictors of axillary pCR. The US-based and combined US-pathology models were developed based on preoperative and postoperative information. The two models had an area under the receiver operating characteristic curve (AUC) of 0.821 and 0.883, respectively, which was significantly higher than that of the fine-needle aspiration model (AUC: 0.735).

**Conclusion:** In this study, based on the US-based model, a combined model incorporating the characteristics of ALNs under US and breast pathology was developed and validated to predict axillary pCR.

## Introduction

Currently, the importance and necessity of neoadjuvant systemic therapy (NST) in the comprehensive treatment of breast cancer are becoming increasingly evident. NST can increase the chance of breast and axillary preservation in patients with breast cancer and provide drug sensitivity testing to guide subsequent adjuvant systemic therapy for patients 1, 2.

Management of the axilla in patients with breast cancer after NST remains controversial. Sentinel lymph node biopsy (SLNB) is generally performed in the axilla after NST in patients with initially clinically negative axillary lymph nodes (cN0), whereas axillary lymph node dissection (ALND) after NST is still the mainstream surgical approach in patients with initially clinically positive axillary lymph nodes (cN+) 3, 4. However, ALND can cause many complications, including numbness of the upper limbs, lymphedema and so on 5. Conservative treatment can be performed when the symptoms are mild, but surgery or even amputation is required in severe cases. Therefore, whether direct ALND is overtreatment in some patients with low axillary lymph node (ALN) tumor load after NST remains unclear. Many prospective clinical trials have investigated the possibility of de-escalation of axillary surgery after NST. The American College of Surgeons Oncology Group Z1071 trial reported that the feasibility of SLNB after NST in patients with cN1 disease was predicated on the resection of lymph nodes containing clip markers identified by blue dye and radiolabeled colloids and the simultaneous detection of at least three lymph nodes 6. However, the high price of clips and the inaccessibility of radiolabeled colloids have limited the application of the results of this study.

In clinical practice, ultrasound (US) is a simple, cost-effective, and highly reproducible examination with high sensitivity, especially in evaluating ALNs 7. Therefore, US examination is a very important component of the comprehensive management of breast cancer patients. Currently, US-guided fine-needle aspiration (FNA) is the most common method for assessing ALN status after NST. However, the clinical applicability of this procedure is low because of its invasiveness and high false-negative rate (FNR) 8, 9. Many previous studies have reported a strong correlation between characteristics of ALNs under US and axillary status in breast cancer, such as blood flow grade, lymphatic hilum structure, lymph node cortical thickness, and longitudinal and transverse diameter ratio 7, 10, 11. Additionally, there is a strong correlation between the efficacy of primary breast tumors after NST and axillary pathological complete response (pCR). Some previous studies have reported that the likelihood of axillary pCR is significantly higher (approximately 90%) if the breast achieved pCR for patients with initially cN+ 12, 13.

Several previous studies have developed models to predict the status of the axilla after NST 14-17. However, all of these models focused only on the clinical and pathological features of the primary breast tumor and ignored the changes in the characteristics of the axillary lymph nodes (ALNs) before and after NST. Therefore, these models have limited accuracy. This study aimed to construct a more accurate combined model incorporating the characteristics of ALNs under US and breast pathology to predict axillary pCR.

## Materials and Methods

### Study design

This retrospective study was approved by the Ethics Review Board of the First Affiliated Hospital of Nanjing Medical University (No. 2023-SR-510). All procedures performed in studies involving human participants were in accordance with the ethical standards of the institutional and national research committee and with the 1964 Helsinki declaration and its later amendments or comparable ethical standards. Due to the retrospective nature of the study, a need for informed consent was waived by the Ethics Review Board.

Data of 657 patients with initially cN+ or pathologically positive axillary lymph nodes who received NST at the Department of Breast Surgery of the First Affiliated Hospital of Nanjing Medical University from 2015 to 2022 were retrospectively collected. In addition, data of 149 patients were diagnosed with axillary metastases by US-guided FNA before NST were also collected, and another FNA was performed after NST (The 149 patients were included in the total of 657 patients). All patients received 6-8 cycles of anthracycline- or paclitaxel-based neoadjuvant regimens, and HER2-positive patients received an additional year of targeted therapy with trastuzumab or trastuzumab combined with pertuzumab. US examination was performed every two cycles (a team of radiologists with more than 5 years of experience in breast ultrasound). The exclusion criteria included (1) patient with cN0, (2) bilateral breast cancer, (3) absence of US examinations before or after NST, (4) absence of pathology after the operation, and (5) concomitant serious diseases, such as severe diabetes, infection, or other malignant tumors. All patients with histologically confirmed ALN metastases or those with ALN metastases considered by US were included in this study.

### Data collection

Data were collected, including age at diagnosis, menopausal status, tumor location, clinical tumor size (cT), clinical lymph nodes (cN), ER status, PR status, HER2 status, Ki-67 expression, US examinations (hilum structure of ALNs, blood flow of ALNs and so on), and pathology after the operation. The US examination was used in Esaote's (Mylab Twice) equipment with a linear transducer from 5 to 13 MHz and Samsung RS80A (Co. Ltd, Seoul, South Korea) equipment with the probe L3-12, 4-13 MHz. Positive ER or PR status were was defined as more than 1% of tumor cells stained positive by immunohistochemistry (IHC) 18. Positive HER2 status was defined as the tumor cells with an IHC score of 3+ or IHC2+/*in situ* hybridization-positive tumor cells 19. The clinical tumor response was evaluated using the Response Evaluation Criteria in Solid Tumors (RECIST) 20. The primary endpoint was axillary pCR, which was defined as the absence of residual invasive carcinoma in ALNs.

### Statistical analysis

All eligible patients were randomly divided into training and validation groups at a ratio of 7:3. The training group was used to develop the model and the validation group was used to validate it. Continuous variables were presented as mean and standard deviation. Categorical variables were presented as frequency and percentage. Between-group differences were analyzed. Variables that were significantly correlated with axillary pCR were examined in the training group using univariate logistic regression analysis. These variables were then subjected to multivariate logistic regression analysis with forward conditional selection. The variables that were finally obtained were independent predictive factors of axillary pCR. These independent factors were used to develop a predictive model. The discriminatory ability, calibration ability, and clinical applicability of the model were evaluated using the receiver operating characteristic (ROC) curve, calibration curve, decision curve analysis (DCA), and clinical impact curve (CIC). The model was validated using data from the validation group. A two-sided *p-*value < 0.05 was considered statistically significant. Odds ratios (ORs) and 95% confidence intervals (CIs) were calculated. All statistical analyses were performed using IBM SPSS Statistics 26.0 software (IBM Corporation, Armonk, NY, USA) and R version 4.3.1 software (The R Foundation for Statistical Computing, Austria, Vienna).

## Results

### Patient characteristics

The study flowchart is shown in Supplementary Figure 1. A total of 657 patients were included in this study. After randomization, 460 patients were included in the training group and 197 in the validation group after randomization. No statistical difference in variables was observed between the training and validation groups (all *p* > 0.05; Supplementary Table 1). Of the 657 patients, 310 (47.1%) achieved axillary pCR. The age at initial diagnosis of patients was approximately 49 years old. Approximately 70% of the patients had cT2 disease. Most of the patients had a significantly smaller primary tumor, and approximately 10% no longer had a visible tumor after NST by US. Approximately 90% of the patients underwent mastectomy. Additionally, 149 patients were diagnosed with axillary metastases by US-guided FNA before NST, and another FNA was performed after NST to clarify the axillary status. Supplementary Table 2 shows the general characteristics of this subset of patients.

### Independent predictors of axillary pCR

**Figure 1:**
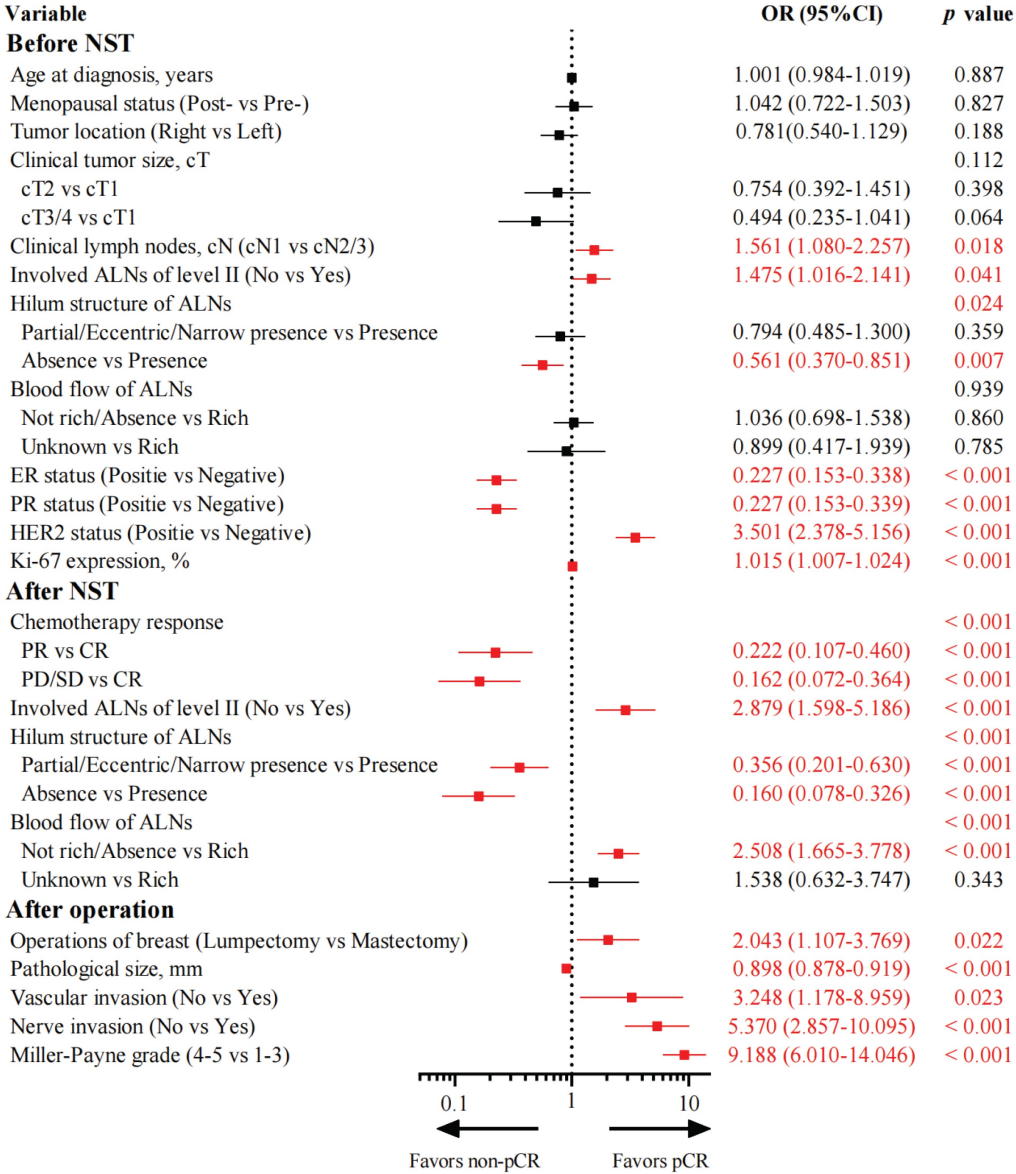
Forest plot of univariate logistic regression analysis of variables associated with axillary pCR in the training group (N = 460). NST, neoadjuvant systemic therapy; ALNs, axillary lymph nodes; CR, complete response; PR, partial response; SD, stable disease; PD, progressive disease; pCR, pathological complete response

Univariate logistic regression analysis clarified 16 variables associated with axillary pCR (Figure 1). Among them, 7 variables were associated with axillary pCR before NST. Patients with cN1 disease (OR: 1.561, 95% CI: 1.080-2.257, *p* = 0.018), no invasion of ALNs of level II (OR: 1.475, 95% CI: 1.016-2.141, *p* = 0.041), positive HER2 status (OR: 3.501, 95% CI: 2.378-5.156, *p* < 0.001), and higher Ki-67 expression (OR: 1.015, 95% CI: 1.007-1.024, *p* < 0.001) were prone to achieve axillary pCR. Patients with positive ER status (OR: 0.227, 95% CI: 0.153-0.338, *p* < 0.001), positive PR status (OR: 0.227, 95% CI: 0.153-0.339, *p* < 0.001), and absence of hilum structure of ALNs (OR: 0.561, 95% CI: 0.370-0.851, *p* = 0.007) were less likely to achieve axillary pCR. Additionally, 4 variables were associated with axillary pCR after NST. Patients with partial response (OR: 0.222, 95% CI: 0.107-0.460, *p* < 0.001) and stable/progressive disease (OR: 0.162, 95% CI: 0.072-0.364, *p* < 0.001) of primary tumors were difficult to achieve axillary pCR compared with those with complete response. Patients with partial/eccentric/narrow presence (OR: 0.356, 95% CI: 0.201-0.630, *p* < 0.001) and absence (OR: 0.160, 95% CI: 0.078-0.326, *p* < 0.001) of hilum structure of ALNs had a smaller probability of achieving axillary pCR than those with the presence of hilum structure of ALNs. Patients who had no invasion of ALNs of level II (OR: 2.879, 95% CI: 1.598-5.186, *p* < 0.001) and had not rich or absent blood flow of ALNs (OR: 2.508, 95% CI: 1.665-3.778, *p* < 0.001) could easily achieve axillary pCR. Furthermore, 5 variables were associated with axillary pCR after the operation. Patients who chose lumpectomy (OR: 2.043, 95% CI: 1.107-3.769, *p* = 0.022), had smaller pathological size (OR: 0.898, 95% CI: 0.878-0.919, *p* < 0.001), had higher Miller-Payne grade (OR: 9.188, 95% CI: 6.010-14.046, *p* < 0.001), and had no vascular (OR: 3.248, 95% CI: 1.178-8.959, *p* = 0.023) or nerve invasion (OR: 5.370, 95% CI: 2.857-10.095, *p* < 0.001) would be preferred to achieve axillary pCR.

Two multivariate logistic regression analyses were performed before and after the operation (Tables 1 and 2). Before the operation, cN (*p* = 0.014), ER status (*p* < 0.001), HER2 status (*p* < 0.001), chemotherapy response of the primary tumor (*p* = 0.011), hilum structure of ALNs after NST (*p* < 0.001), and blood flow of ALNs after NST (*p* = 0.004) were independent predictive factors of axillary pCR. After the operation, cN (*p* = 0.003), ER status (*p* < 0.001), HER2 status (*p* = 0.009), hilum structure of ALNs after NST (*p* < 0.001), blood flow of ALNs after NST (*p* = 0.003), vascular invasion (*p* = 0.043), pathological size (*p* = 0.001), and Miller-Payne grade (*p* = 0.006) were independent predictive factors of axillary pCR.

### Development and validation of the US-based model

**Figure 2:**
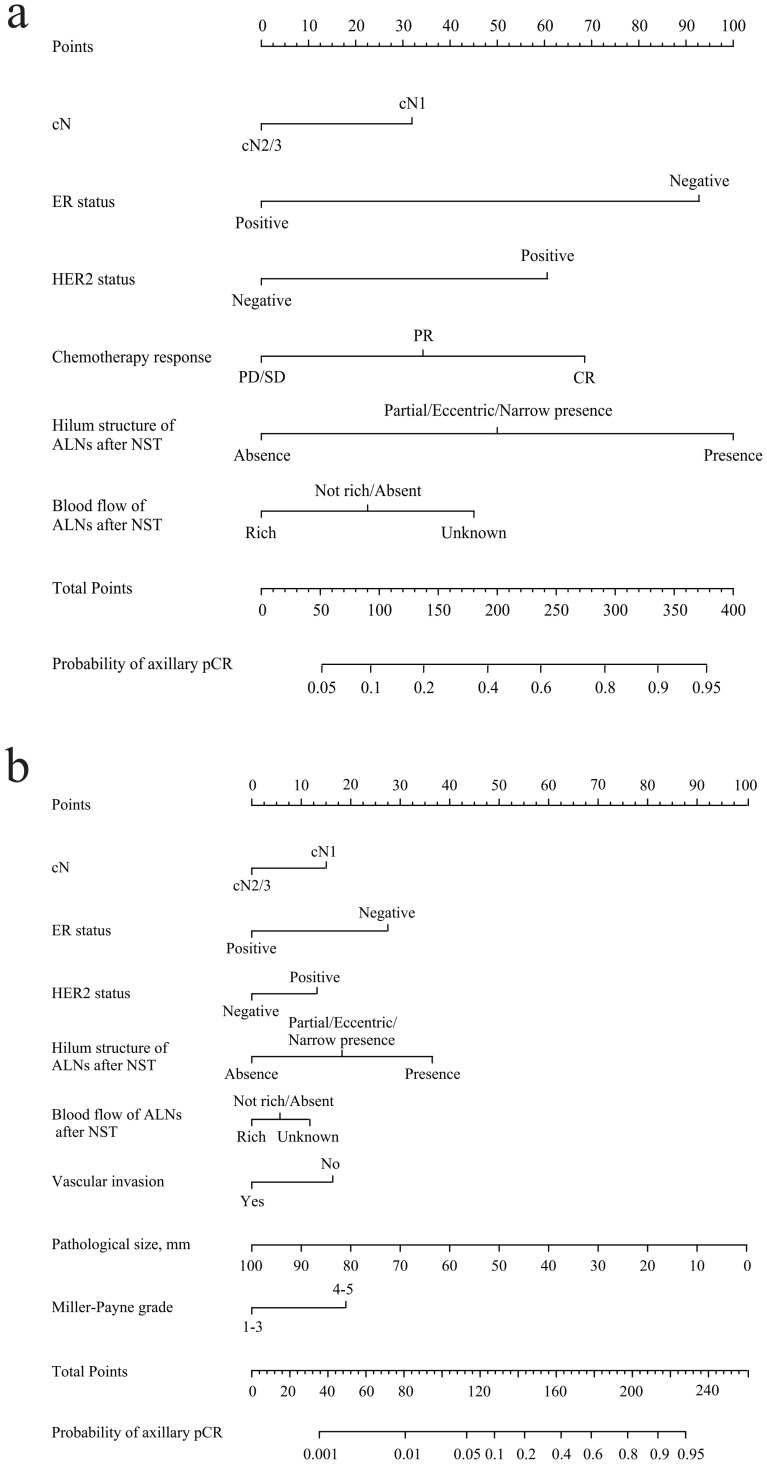
**(a)** Preoperative US-based nomogram model. **(b)** Postoperative combined US-pathology nomogram model. Each point that corresponds to each variable is on the uppermost point scale. The sum of all points is the total points. The total points projected at the bottom scale indicate the probability of axillary pCR. cN, clinical lymph nodes; CR, complete response; PR, partial response; SD, stable disease; PD, progressive disease; ALNs, axillary lymph nodes; pCR, pathological complete response.

**Figure 3:**
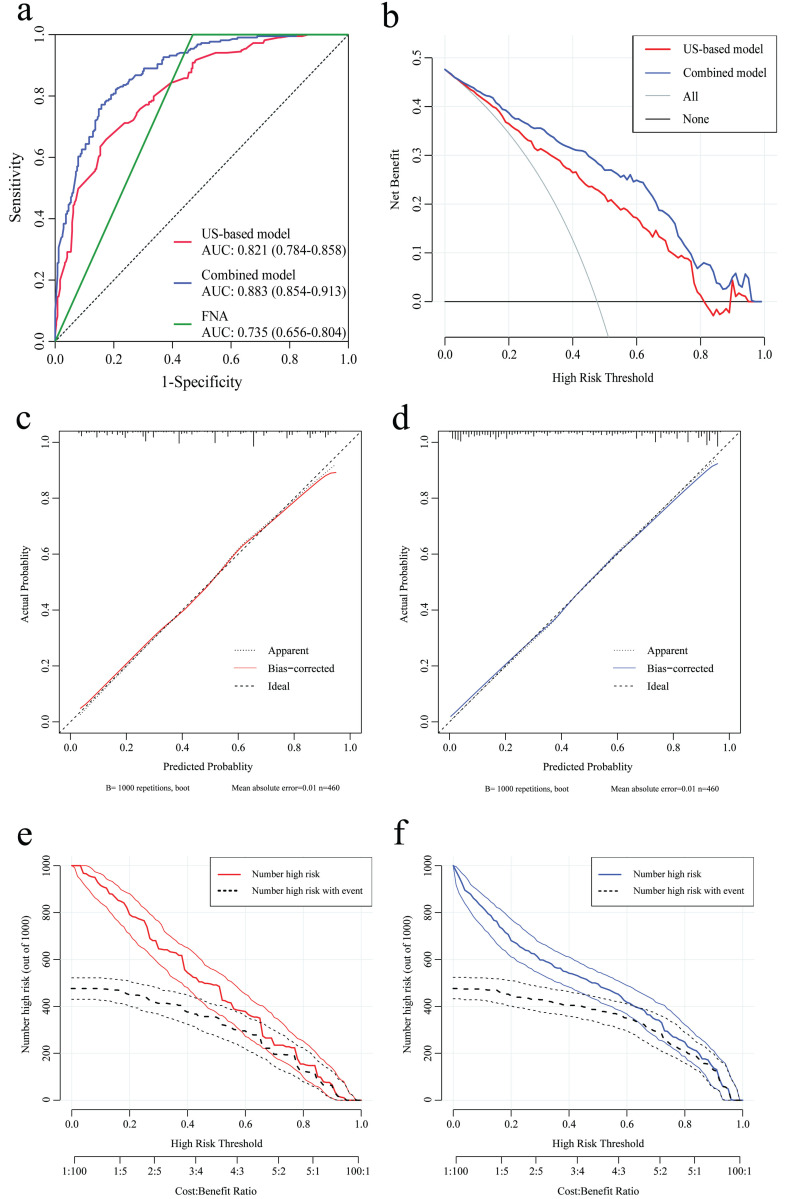
The receiver operating characteristic (ROC) curve, decision curve analysis (DCA), calibration curve, and clinical impact curve (CIC) of the US-based and combined US-pathology nomogram models in the training group. **(a)** The area under the receiver operating characteristic curve (AUC) of the US-based, combined, and FNA models are 0.821 (0.784-0.858), 0.883 (0.854-0.913), and 0.735 (0.656-0.804), respectively. **(b)** DCA for the US-based (red) and combined (blue) models in the training group. The red and blue lines represent the two models. The gray line represents the assumption that all patients were responders. The black line represents the hypothesis that no patients were responders. **(c and d)** Calibration curves of the US-based model (c) and the combined model (d) in the training group. In the calibration curves, a dotted line at a 45° angle represents perfect calibration. **(e and f)** CIC for the US-based model (e) and the combined model (f) in the training group. CIC showed the model's estimated number that would be declared high risk for each risk threshold and the proportion of true positive patients. FNA, fine-needle aspiration.

According to the results of multivariate analysis before the operation, a nomogram model based on US characteristics was developed to predict the probability of axillary pCR (Figure 2a). The probability of axillary pCR should be evaluated preoperatively based on the US characteristics of a patient before and after NST and the IHC results. The US-based model had an area under the receiver operating characteristic curve (AUC) of 0.821 (0.784-0.858) in the training group and 0.844 (0.790-0.898) in the validation group, whose diagnostic power was significantly higher than that of the FNA model [AUC: 0.735 (0.656-0.804), *p* = 0.010] (Figure 3a and Supplementary Figure 2a). The calibration was good for the training and validation groups. No significant difference was observed between the predicted and actual probabilities of achieving axillary pCR, indicating that the US-based model was well calibrated (Figure 3c and Supplementary Figure 2c). The clinical applicability of the US-based model was assessed using DCA and CIC. The clinical net benefit of the US-based model was assessed using DCA, which revealed that the model would provide a clinical net benefit to a vast majority of the patients in a wide threshold range (0-80%) in the training and validation groups (Figure 3b and Supplementary Figure 2b). The CIC showed that the “Number high risk” curve was close to the “Number high risk with the event” curve at the high-risk threshold of 0.4-1 in the training and validation groups (Figure 3e and Supplementary Figure 2e). The CIC intuitively showed that the nomogram provided a superior overall net benefit based on the practical ranges of threshold probabilities, indicating that the model possessed significant predictive value for identifying patients with axillary pCR.

### Development and validation of the combined US-pathology model

**Figure 4:**
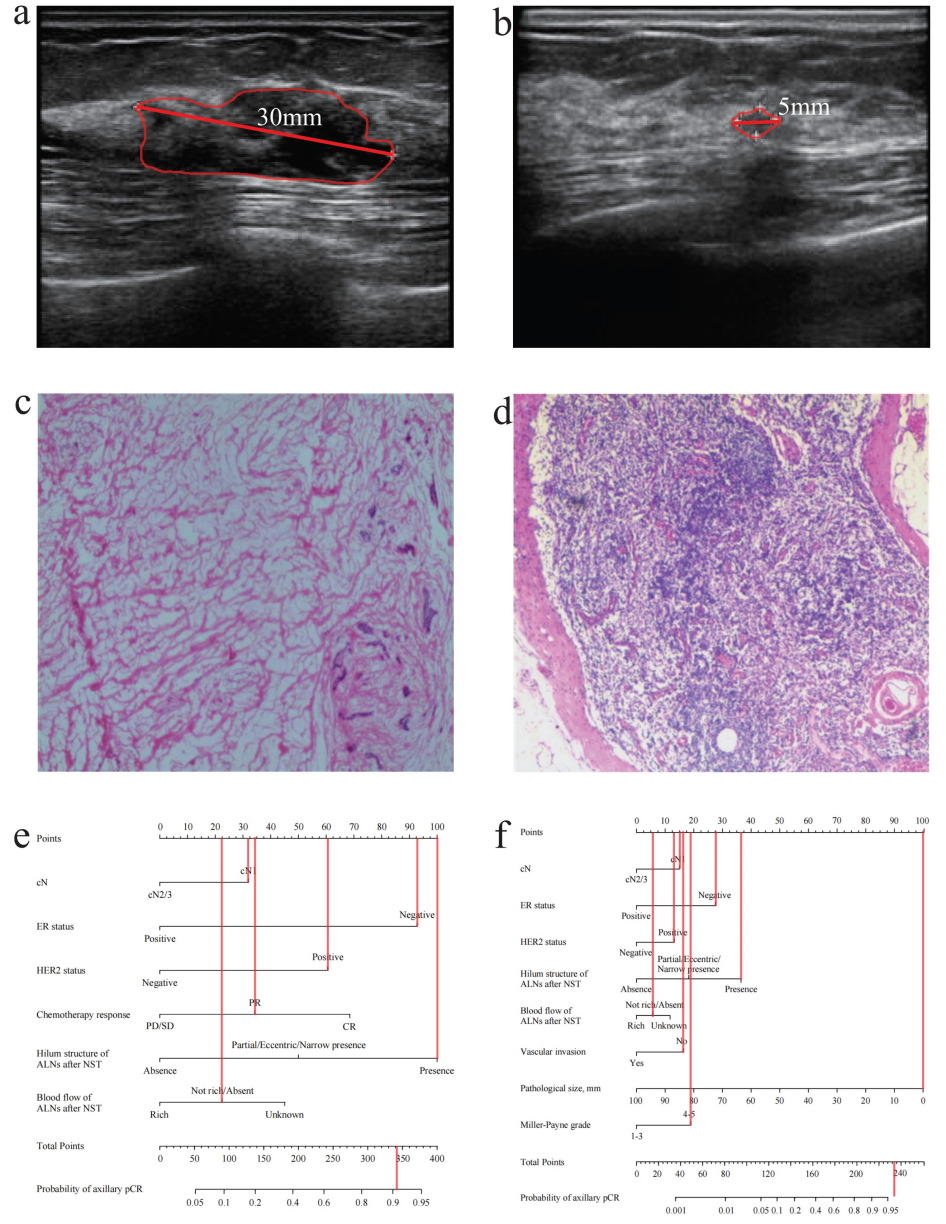
A patient with axillary pCR (cN1, negative ER status, positive HER2 status, present hilum structure of ALNs after NST, absent blood flow of ALNs after NST, no vascular invasion, pathological size: 0mm, Miller-Payne grade: 5). **(a)** The ultrasound before NST (the diameter of tumor: 30mm). **(b)** The ultrasound after NST (the diameter of tumor: 5mm).** (c)** The pathology of breast after operation (breast pCR). **(d)** The pathology of ALNs after NST (axillary pCR). **(e)** The probability of axillary pCR was approximately 90% based on the US-based model. **(f)** The probability of axillary pCR was over 95% based on the combined model. pCR, pathological complete response; cN, clinical lymph nodes; ALNs, axillary lymph nodes; NST, neoadjuvant systemic therapy; US, ultrasound.

According to the results of multivariate analysis after the operation, a combined model based on clinical, US, and pathological characteristics was developed (Figure 2b). The combined model had an AUC of 0.883 (0.854-0.913) in the training group and 0.907 (0.847-0.967) in the validation group, which was significantly higher than that in the US-based model (*p* < 0.001) and FNA model (*p* < 0.001) (Figure 3a and Supplementary Figure 2a). Additionally, the calibration ability of the combined model was better than that of the US-based model (Figure 3d and Supplementary Figure 2d). The DCA and CIC of the combined model also showed superior clinical applicability to the US-based model (Figures 3b, 3f, and Supplementary Figures 2b, and 2f). Figure 4 showed a representative example of a patient with a probability of predicting axillary pCR of about 90% based on the US-based model and over 95% based on the combined model.

## Discussion

In the current study, the data of 657 patients with breast cancer who underwent NST at our center were retrospectively analyzed. Based on the results of univariate and multivariate logistic analyses, nomogram models were developed to predict axillary pCR after NST in the preoperative and postoperative periods, respectively.

Overall, the axillary pCR rate was 47.1% after the operation, which is close to that reported in the previous clinical studies 17, 21, 22. In clinical practice, performing FNA is the most common way to clarify the status of ALNs before the operation. However, this method has major drawbacks, such as invasiveness and a high FNR. In our center, we previously attempted to clarify the ALNs status by FNA preoperatively. However, the FNR of this method was too high (FNR: 53.6%), and the accuracy was too low (AUC: 0.735), which was similar to previous studies 8, 9. This may be mainly due to NST-induced fibrosis of lymph nodes and lymphatic vessels, resulting in the inability to accurately identify positive lymph nodes preoperatively 23.

The pCR rate after NST for different molecular subtypes of breast cancer tended to be different. Because of the insensitivity of luminal breast cancer to chemotherapy, triple-negative and HER2-positive breast cancers showed higher pCR rates than luminal breast cancers. The multivariate logistic regression analysis showed that HER2 status and ER status were independent influencing factors for axillary pCR. This result is consistent with that of previous clinical trials 14, 16, 24. US has a unique advantage in the evaluation of ALNs in patients with breast cancer, thus playing a significant role during NST 25, 26. Therefore, a US-based predictive model was developed to more precisely identify the ALN status after NST. In addition to the subtypes of breast cancer, the hilum structure of ALNs after NST and blood flow of ALNs after NST were independent predictors of pCR in ALNs. Previous studies have shown that the absence of hilum structure of ALNs in patients with breast cancer or the richness of blood flow of ALNs indicates cancer metastases, which is consistent with our findings 27, 28. Validation was performed, and it was found that this noninvasive US-based model had an AUC of 0.821.

Some previous studies have developed predictive models based on US to predict pCR after NST. However, these models focused only on some indicators under US and ignored pathological changes in the breast 29, 30. The predictive ability of these models was not high. Several previous clinical studies have reported that changes in the efficacy of the primary breast tumors before and after NST could more accurately predict the axillary response to NST 31, 32. Therefore, data on the pathology of breast tumors were retrospectively collected and analyzed. The results showed that many indicators associated with breast pathology were independent predictors of axillary pCR, such as vascular invasion, pathological size, and Miller-Payne grade of the primary tumor. The above three predictors represent the efficacy response of the primary breast tumor to NST. Furthermore, it has been reported that if the primary breast tumor shrinks significantly after NST or even if the breast achieves pCR, the likelihood of achieving pCR in ALNs is high 13. Based on the results of a previous study at our center, among those with initial cN+, the rate of axillary pCR was higher in the breast pCR group than in the breast non-pCR group (82.7% vs. 22.9%, *p* < 0.0001) 33. Therefore, accurately identifying whether the breast achieves pCR preoperatively is the biggest clinical challenge.

A clinical trial from MD Anderson Cancer Center reported the first oncologic outcomes for the omission of breast surgery using a vacuum-assisted biopsy (VAB) performed after NST in patients with strict inclusion criteria (cT1-2, cN0-1, triple-negative or HER2-positive breast cancers, residual lesion < 2 cm on imaging after NST). At a median follow-up of 26.4 months, no ipsilateral breast tumor recurrences occurred in 31 eligible patients 34. Another multicenter clinical trial that assessed the accuracy of post-NST image-guided VAB in predicting residual cancer in the breast showed that a standardized protocol using image-guided VAB of a tumor bed measuring 2 cm or smaller with 6 or more representative samples allowed reliable prediction of residual disease (FNR: 3.2%) 35. These findings indicate that, with the development of precision medicine and personalized oncology therapy, it is likely that the assessment of breast pCR can be achieved preoperatively, especially in triple-negative and HER2-positive breast cancers, where pCR is more easily achieved.

Recently, some constructed nomogram models have incorporated US or MRI features and used texture analysis or machine learning-based radiomics, demonstrating higher predictive performance 36, 37. However, texture analysis and radiomics are not easy to apply in clinical practice, and reproducibility may be low. The US variables used in our study are relatively simple and easy to apply without dedicated software or postprocessing. Therefore, our model has higher clinical applicability and reproducibility than machine learning-based radiomics.

However, there were some limitations in this study. First, this was a single-center retrospective clinical study. There may be a risk of data loss and selection bias in the collection of patient data because those patients without complete US and pathology data were excluded. Second, the model was internally validated, and the study had a small sample size in the validation group. Therefore, further studies with large samples that include data from other centers are needed to externally validate the model. Finally, this study did not evaluate US features using texture analysis or machine learning-based radiomics. Thus, further studies are needed to assess whether clinicopathological features combined with radiomics to construct models could further improve the predictive performance of the models.

## Conclusion

In conclusion, based on the US-based model, a combined model incorporating the characteristics of ALNs under US and breast pathology is developed to predict axillary pCR, and the combined US-pathology model is validated to have considerable accuracy. The combined model will be useful in designing clinical trials to screen out patients who may achieve axillary pCR while attempting to perform axillary de-escalation surgery.

## Supplementary Material

Supplementary figures and tables.

## Figures and Tables

**Table 1:** Multivariate logistic regression analysis of variables associated with axillary pCR before the operation.

Variable	OR	95% CI	*p* value
Clinical lymph nodes, cN			
cN1 vs cN2/3	1.769	1.121-2.792	0.014
ER status			
Positive vs Negative	0.168	0.104-0.273	< 0.001
HER2 status			
Positive vs Negative	3.079	1.960-4.838	< 0.001
Chemotherapy response			0.011
PR vs CR	0.248	0.096-0.640	0.004
PD/SD vs CR	0.297	0.128-0.689	0.005
Hilum structure of ALNs after NST			< 0.001
Partial/Eccentric/Narrow presence vs Presence	0.410	0.208-0.808	0.010
Absence vs Presence	0.175	0.077-0.395	< 0.001
Blood of ALNs after NST			0.004
Not rich/Absent vs Rich	2.202	1.340-3.617	0.002
Unknown vs Rich	0.880	0.308-2.519	0.812

Abbreviations: CR, complete response; PR, partial response; SD, stable disease; PD, progressive disease; ALNs, axillary lymph nodes

**Table 2:** Multivariate logistic regression analysis of variables associated with axillary pCR after the operation.

Variable	OR	95% CI	*p* value
Clinical lymph nodes, cN			
cN1 vs cN2/3	2.160	1.293-3.608	0.003
ER status			
Positive vs Negative	0.218	0.127-0.373	< 0.001
HER2 status			
Positive vs Negative	1.987	1.191-3.317	0.009
Hilum structure of ALNs after NST			< 0.001
Partial/Eccentric/Narrow presence vs Presence	0.392	0.184-0.835	0.015
Absence vs Presence	0.165	0.067-0.410	< 0.001
Blood of ALNs after NST			0.003
Not rich/Absent vs Rich	2.212	1.276-3.833	0.005
Unknown vs Rich	0.591	0.196-1.782	0.350
Pathological size, mm	0.949	0.921-0.979	0.001
Vascular invasion			
No vs Yes	2.253	1.025-4.952	0.043
Miller-Payne grade			
4-5 vs 1-3	2.659	1.331-5.310	0.006

Abbreviations: ALNs, axillary lymph nodes

## References

[1] Waks AG, Winer EP (2019). Breast Cancer Treatment: A Review. JAMA.

[2] Steenbruggen TG, van Ramshorst MS, Kok M, Linn SC, Smorenburg CH, Sonke GS (2017). Neoadjuvant Therapy for Breast Cancer: Established Concepts and Emerging Strategies. Drugs.

[3] Boland MR, Al-Hilli Z (2021). Management of the axilla after neoadjuvant chemotherapy in breast cancer patients. Br J Surg.

[4] Pilewskie M, Morrow M (2017). Axillary Nodal Management Following Neoadjuvant Chemotherapy: A Review. JAMA Oncol.

[5] Cocco D, Shah C, Wei W, Wilkerson A, Grobmyer SR, Al-Hilli Z (2022). Axillary lymph node dissection can be omitted in patients with limited clinically node-positive breast cancer: a National Cancer Database analysis. Br J Surg.

[6] Boughey JC, Suman VJ, Mittendorf EA, Ahrendt GM, Wilke LG, Taback B (2013). Sentinel lymph node surgery after neoadjuvant chemotherapy in patients with node-positive breast cancer: the ACOSOG Z1071 (Alliance) clinical trial. JAMA.

[7] Ferroni G, Sabeti S, Abdus-Shakur T, Scalise L, Carter JM, Fazzio RT (2023). Noninvasive prediction of axillary lymph node breast cancer metastasis using morphometric analysis of nodal tumor microvessels in a contrast-free ultrasound approach. Breast cancer research: BCR.

[8] Wu S-Y, Li J-W, Wu H-L, Shao Z-M, Liu G-Y, Hu N (2022). Accuracy of ultrasound-guided targeted fine-needle aspiration in asses sing nodal response in node-positive breast cancer after neoadjuvant c hemotherapy: prospective feasibility study. Br J Surg.

[9] Caudle AS, Kuerer HM, Krishnamurthy S, Shin K, Hobbs BP, Ma J (2019). Feasibility of fine-needle aspiration for assessing responses to chemotherapy in metastatic nodes marked with clips in breast cancer: A prospective registry study. Cancer.

[10] Zhang H, Cao W, Liu L, Meng Z, Sun N, Meng Y (2023). Noninvasive prediction of node-positive breast cancer response to presurgical neoadjuvant chemotherapy therapy based on machine learning of axillary lymph node ultrasound. Journal of translational medicine.

[11] Zhu Y, Jia Y, Pang W, Duan Y, Chen K, Nie F (2023). Ultrasound contrast-enhanced patterns of sentinel lymph nodes: predictive value for nodal status and metastatic burden in early breast cancer. Quantitative imaging in medicine and surgery.

[12] Choi HJ, Ryu JM, Kim I, Nam SJ, Kim SW, Yu J (2019). Prediction of axillary pathologic response with breast pathologic complete response after neoadjuvant chemotherapy. Breast Cancer Res Treat.

[13] Tadros AB, Yang WT, Krishnamurthy S, Rauch GM, Smith BD, Valero V (2017). Identification of Patients With Documented Pathologic Complete Response in the Breast After Neoadjuvant Chemotherapy for Omission of Axillary Surgery. JAMA Surg.

[14] Wang W, Wang X, Liu J, Zhu Q, Wang X, Wang P (2021). Nomogram for predicting axillary lymph node pathological response in node-positive breast cancer patients after neoadjuvant chemotherapy. Chinese medical journal.

[15] Guo R, Su Y, Si J, Xue J, Yang B, Zhang Q (2020). A nomogram for predicting axillary pathologic complete response in hormone receptor-positive breast cancer with cytologically proven axillary lymph node metastases. Cancer.

[16] Shi W, Huang X, Wang Y, Wan X, He J, Xu Y (2022). A novel nomogram containing efficacy indicators to predict axillary pathologic complete response after neoadjuvant systemic therapy in breast cancer. Front Endocrinol (Lausanne).

[17] Corsi F, Albasini S, Sorrentino L, Armatura G, Carolla C, Chiappa C (2021). Development of a novel nomogram-based online tool to predict axillary status after neoadjuvant chemotherapy in cN+ breast cancer: A multicentre study on 1,950 patients. Breast (Edinburgh, Scotland).

[18] Hammond MEH, Hayes DF, Dowsett M, Allred DC, Hagerty KL, Badve S (2010). American Society of Clinical Oncology/College Of American Pathologists guideline recommendations for immunohistochemical testing of estrogen and progesterone receptors in breast cancer. J Clin Oncol.

[19] Wolff AC, Hammond MEH, Allison KH, Harvey BE, Mangu PB, Bartlett JMS (2018). Human Epidermal Growth Factor Receptor 2 Testing in Breast Cancer: American Society of Clinical Oncology/College of American Pathologists Clinical Practice Guideline Focused Update. J Clin Oncol.

[20] Eisenhauer EA, Therasse P, Bogaerts J, Schwartz LH, Sargent D, Ford R (2009). New response evaluation criteria in solid tumours: revised RECIST guideline (version 1.1). Eur J Cancer.

[21] Gerber B, Schneeweiss A, Möbus V, Golatta M, Tesch H, Krug D (2022). Pathological Response in the Breast and Axillary Lymph Nodes after Neoadjuvant Systemic Treatment in Patients with Initially Node-Positive Breast Cancer Correlates with Disease Free Survival: An Exploratory Analysis of the GeparOcto Trial. Cancers (Basel).

[22] Zhao F, Miyashita M, Hattori M, Yoshimatsu T, Howard F, Kaneva K (2023). Racial Disparities in Pathological Complete Response Among Patients Receiving Neoadjuvant Chemotherapy for Early-Stage Breast Cancer. JAMA network open.

[23] Kuerer HM, Rauch GM, Krishnamurthy S, Adrada BE, Caudle AS, DeSnyder SM (2018). A Clinical Feasibility Trial for Identification of Exceptional Responders in Whom Breast Cancer Surgery Can Be Eliminated Following Neoadjuvant Systemic Therapy. Ann Surg.

[24] Malhaire C, Selhane F, Saint-Martin MJ, Cockenpot V, Akl P, Laas E (2023). Exploring the added value of pretherapeutic MR descriptors in predicting breast cancer pathologic complete response to neoadjuvant chemotherapy. European radiology.

[25] Huang JX, Lin SY, Ou Y, Shi CG, Zhong Y, Wei MJ (2022). Combining conventional ultrasound and sonoelastography to predict axillary status after neoadjuvant chemotherapy for breast cancer. European radiology.

[26] Maeshima Y, Sakai T, Ogiya A, Takahashi Y, Miyagi Y, Kokubu Y (2021). Assessment of axillary node status by ultrasound after neoadjuvant chemotherapy in patients with clinically node-positive breast cancer according to breast cancer subtype. Sci Rep.

[27] Xu H, Xu GL, Li XD, Su QH, Dong CZ (2021). Correlation between the contrast-enhanced ultrasound image features an d axillary lymph node metastasis of primary breast cancer and its diag nostic value. Clin Transl Oncol.

[28] Gu L-S, Zhang R, Wang Y, Liu X-M, Ma F, Wang J-Y (2019). Characteristics of contrast-enhanced ultrasonography and strain elasto graphy of locally advanced breast cancer. J Thorac Dis.

[29] Skarping I, Förnvik D, Zackrisson S, Borgquist S, Rydén L (2021). Predicting pathological axillary lymph node status with ultrasound following neoadjuvant therapy for breast cancer. Breast Cancer Res Treat.

[30] Pislar N, Gasljevic G, Music MM, Borstnar S, Zgajnar J, Perhavec A (2023). Axillary ultrasound for predicting response to neoadjuvant treatment in breast cancer patients-a single institution experience. World journal of surgical oncology.

[31] Barron AU, Hoskin TL, Day CN, Hwang ES, Kuerer HM, Boughey JC (2018). Association of Low Nodal Positivity Rate Among Patients With ERBB2-Positive or Triple-Negative Breast Cancer and Breast Pathologic Complete Response to Neoadjuvant Chemotherapy. JAMA Surg.

[32] Samiei S, van Nijnatten TJA, de Munck L, Keymeulen K, Simons JM, Kooreman LFS (2020). Correlation Between Pathologic Complete Response in the Breast and Absence of Axillary Lymph Node Metastases After Neoadjuvant Systemic Therapy. Ann Surg.

[33] Chen R, Li S, Li Y, Zhu Q, Shi X, Xu L (2021). Can axillary surgery be omitted in patients with breast pathologic complete response after neoadjuvant systemic therapy for breast cancer? A real-world retrospective study in China. J Cancer Res Clin Oncol.

[34] Kuerer HM, Smith BD, Krishnamurthy S, Yang WT, Valero V, Shen Y (2022). Eliminating breast surgery for invasive breast cancer in exceptional responders to neoadjuvant systemic therapy: a multicentre, single-arm, phase 2 trial. Lancet Oncol.

[35] Tasoulis MK, Lee HB, Yang W, Pope R, Krishnamurthy S, Kim SY (2020). Accuracy of Post-Neoadjuvant Chemotherapy Image-Guided Breast Biopsy to Predict Residual Cancer. JAMA Surg.

[36] Gu J, Tong T, Xu D, Cheng F, Fang C, He C (2023). Deep learning radiomics of ultrasonography for comprehensively predicting tumor and axillary lymph node status after neoadjuvant chemotherapy in breast cancer patients: A multicenter study. Cancer.

[37] Liu S, Du S, Gao S, Teng Y, Jin F, Zhang L (2023). A delta-radiomic lymph node model using dynamic contrast enhanced MRI for the early prediction of axillary response after neoadjuvant chemotherapy in breast cancer patients. BMC Cancer.

