# Supplementary material for: Development and validation of a combined ultrasound-pathology model to predict axillary status after neoadjuvant systemic therapy in breast cancer
Source: Int J Med Sci. 2024 Oct 21;21(14):2714–24. doi: 10.7150/ijms.101855 (PMC11539384; doi:10.7150/ijms.101855)
Supplement: Supplementary file 1 — Supplementary figures and tables. [file ijmsv21p2714s1.pdf]

Supplementary Table 1. The characteristics of patients before NST, after NST, and after the operation.

Characteristics	Training group N=460, %	Validation group N=197, %	*p* value
**Before NST**			
Age at diagnosis, year	49.0 ± 10.4	48.9 ± 9.9	0.931
Menopausal status			0.675
Pre-menopausal	244 (53.0)	108 (54.8)	
Post-menopausal	216 (47.0)	89 (45.2)	
Tumor location			0.286
Left breast	252 (54.8)	99 (50.3)	
Right breast	208 (45.2)	98 (49.7)	
Clinical tumor size, cT			0.981
cT1	41 (8.9）	17 (8.6)	
cT2	326 (70.9)	139 (70.6)	
cT3/4	93 (20.2)	41 (20.8)	
Clinical lymph nodes, cN			0.187
cN1	243 (52.8)	93 (47.2)	
cN2/3	217 (47.2)	104 (52.8)	
Involved ALNs of level II			0.585
Yes	195 (42.4)	79 (40.1)	
No	265 (57.6)	118 (59.9)	
Hilum structure of ALNs			0.887
Presence	192 (41.7)	81 (41.1)	
Partial/Eccentric/Narrow presence	95 (20.7)	44 (22.3)	
Absence	173 (37.6)	72 (36.5)	
Blood of ALNs			0.474
Rich	278 (60.4)	129 (65.5)	
Not rich/Absent	153 (33.3)	57 (28.9)	
Unknown	29 (6.3)	11 (5.6)	
ER status			0.774
Positive	270 (58.7)	118 (59.9)	
Negative	190 (41.3)	79 (40.1)	
PR status			0.555
Positive	201 (43.7)	91 (46.2)	

Negative	259 (56.3)	106 (53.8)	
HER2 status			0.453
Positive	199 (43.3)	79 (40.1)	
Negative	261 (56.7)	118 (59.9)	
Ki-67 expression, %	48.3 ± 22.2	46.4 ± 22.3	0.338
**After NST**			
Clinical tumor response			0.266
CR	48 (10.4)	19 (9.6)	
PR	315 (68.5)	125 (63.5)	
PD/SD	97 (21.1)	53 (26.9)	
Involved ALNs of level II			0.675
Yes	64 (13.9)	25 (12.7)	
No	396 (86.1)	172 (87.3)	
Hilum structure of ALNs			0.775
Presence	337 (73.3)	145 (73.6)	
Partial/Eccentric/Narrow presence	64 (13.9)	24 (12.2)	
Absence	59 (12.8)	28 (14.2)	
Blood of ALNs			0.840
Rich	153 (33.3)	64 (32.5)	
Not rich/Absent	284 (61.7)	125 (63.5)	
Unknown	23 (5.0)	8 (4.1)	
**After operation**			
Operations of breast			0.848
Mastectomy	411 (89.3)	177 (89.8)	
Lumpectomy	49 (10.7)	20 (10.2)	
Pathological size, mm	13.7 ± 16.2	15.3 ± 16.5	0.241
Vascular invasion			0.398
Yes	74 (16.1)	37 (18.8)	
No	386 (83.9)	160 (81.2)	
Nerve invasion			0.488
Yes	22 (4.8)	12 (6.1)	
No	438 (95.2)	185 (93.9)	
Miller-Payne grade			0.601
1-3	249 (54.1)	111 (56.3)	

4-5	211 (45.9)	86 (43.7)

Abbreviations: ALNs, axillary lymph nodes; CR, complete response; PR, partial response; SD, stable disease; PD, progressive disease

Supplementary Table 2. The characteristics of pathologically-node positive patients who received FNA after NST.

Characteristics	FNA (-) N=110, %	FNA (+) N=39, %	*p* value
Age at diagnosis, year	48.9 ± 10.2	48.7 ± 9.0	0.929
Menopausal status			0.565
Pre-menopausal	59 (53.6)	23 (59.0)	
Post-menopausal	51 (46.4)	16 (41.0)	
Tumor location			0.281
Left breast	70 (63.6)	21 (53.8)	
Right breast	40 (36.4)	18 (46.2)	
Clinical tumor size (cT)			0.938
cT1	8 (7.3)	2 (5.1)	
cT2	80 (72.7)	29 (74.4)	
cT3	13 (11.8)	4 (10.3)	
cT4	9 (8.2)	4 (10.3)	
Clinical lymph nodes (cN)			0.950
cN1	60 (54.5)	22 (56.4)	
cN2	34 (30.9)	11 (28.2)	
cN3	16 (14.5)	6 (15.4)	
ER status			0.307
Positive	69 (62.7)	28 (71.8)	
Negative	41 (37.3)	11 (28.2)	
PR status			0.281
Positive	51 (46.4)	22 (56.4)	
Negative	59 (53.6)	17 (43.6)	
HER2 status			< 0.001
Positive	65 (59.1）	9 (23.1)	
Negative	45 (40.9)	30 (76.9)	
Ki-67 expression, %	46.2 ± 21.1	42.4 ± 23.5	0.344
Clinical tumor response			0.003
CR	16 (14.5)	0 (0)	
PR	76 (69.1)	24 (61.5)	
SD	16 (14.5)	15 (38.5)	
PD	2 (1.8)	0 (0)	

Operations of breast			0.015
Mastectomy	95 (86.4)	39 (100)	
Lumpectomy	15 (13.6)	0 (0)	
Pathological size, mm	9.8 ± 13.7	24.2 ± 19.5	< 0.001
Vascular invasion			0.011
Yes	14 (12.7)	12 (30.8)	
No	96 (87.3)	27 (69.2)	
Nerve invasion			0.684
Yes	4 (3.6)	2 (5.1)	
No	106 (96.4)	37 (94.9)	
Miller-Payne grade			< 0.001
1-3	42 (38.2)	32 (82.1)	
4-5	68 (61.8)	7 (17.9)	
Axillary pCR			< 0.001
Yes	66 (60.0)	0	
No	44 (40.0)	39 (100)	

Abbreviations: FNA, fine-needle aspiration; ALNs, axillary lymph nodes; CR, complete response; PR, partial response; SD, stable disease; PD, progressive disease

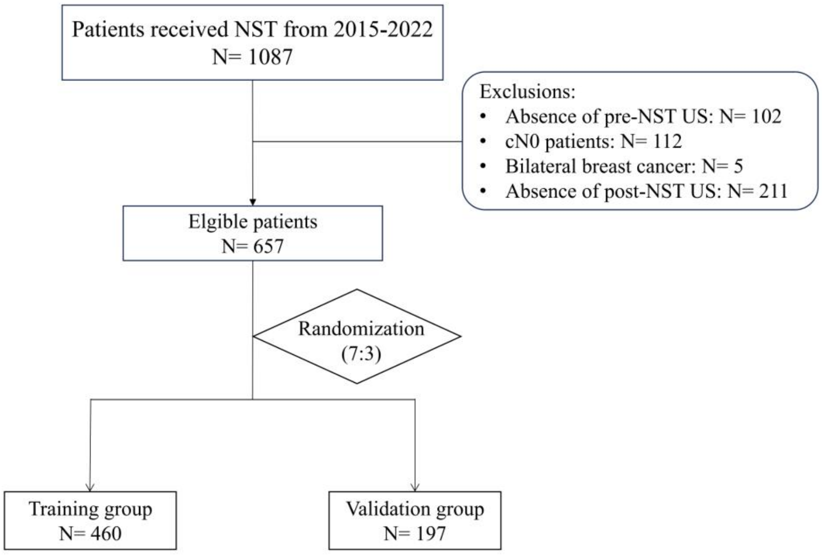

Supplementary Figure 1. Study flowchart. NST, neoadjuvant systemic therapy; US, ultrasound; cN0, clinically negative axillary lymph nodes

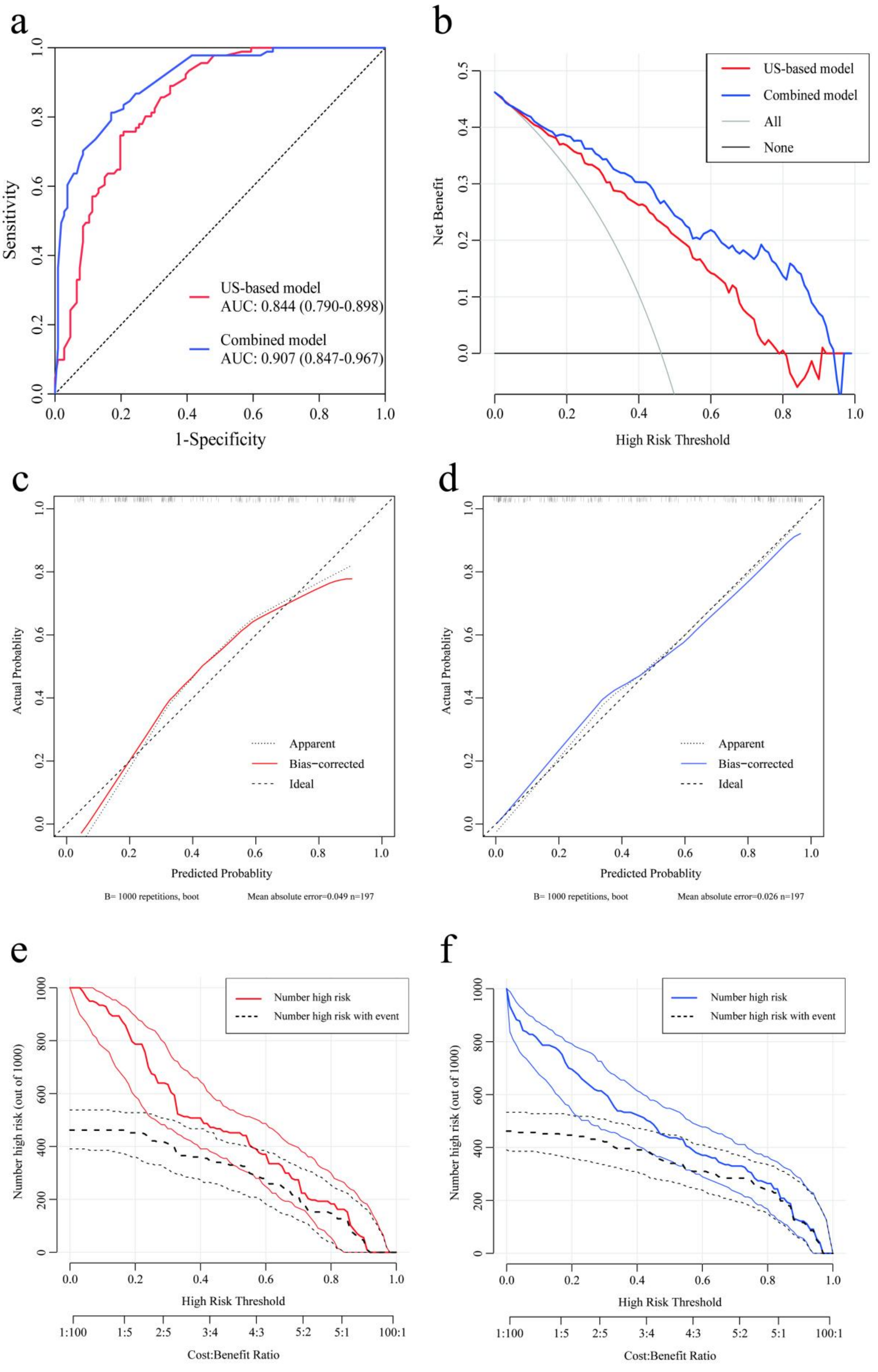

Supplementary Figure 2. The receiver operating characteristic (ROC) curve, decision curve analysis (DCA), calibration curve, and clinical impact curve (CIC) of the US-based and combined US-pathology nomogram models in the validation group. (a) The area under the receiver operating characteristic curve (AUC) of the US-based

and combined models are 0.844 (0.790–0.898) and 0.907 (0.847–0.967), respectively. (b) DCA for the US-based (red) and combined (blue) models in the validation group. The red and blue lines represent the two models. The gray line represents the assumption that all patients were responders. The black line represents the hypothesis that no patients were responders. (c and d) Calibration curves of the US-based model (c) and the combined model (d) in the validation group. In the calibration curves, a dotted line at a 45° angle represents perfect calibration. (e and f) CIC for the US-based model (e) and the combined model (f) in the validation group. CIC showed the model's estimated number that would be declared high risk for each risk threshold and the proportion of true positive patients.
